# Intravoxel Incoherent Motion Diffusion-Weighted MR Imaging for Monitoring the Immune Response of Immunogenic Chemotherapy

**DOI:** 10.3389/fonc.2022.796936

**Published:** 2022-05-13

**Authors:** Junjiao Hu, Xin Yu, Peidi Yin, Bin Du, Xiangran Cai

**Affiliations:** ^1^ Medical Imaging Center, First Affiliated Hospital of Jinan University, Guangzhou, China; ^2^ Medical Imaging Center, Shenzhen Hospital, Southern Medical University, Shenzhen, China; ^3^ Department of Pathology, First Affiliated Hospital of Jinan University, Guangzhou, China; ^4^ Department of Pathology, Shanghai First Maternity and Infant Hospital, Tongji University School of Medicine, Shanghai, China

**Keywords:** magnetic resonance imaging, diffusion-weighted imaging, intravoxel incoherent motion, metronomic chemotherapy, immune response prediction

## Abstract

**Objective:**

To evaluate the predictive value of intravoxel incoherent motion (IVIM) diffusion-weighted imaging (DWI) in the quantitative assessment of conventional chemotherapy-activated immune responses in mouse tumor models and clinics.

**Methods:**

A total of 19 subcutaneous tumor-bearing mice were randomly divided into treated and control groups. Both groups had orderly IVIM DWI examinations before and on days 6 and 12 after the administration of cyclophosphamide (CPA) or saline. Pathologic examinations were performed, including HE staining and immunohistochemistry (IHC). The expressions of immune-related genes in the tumor were measured by qPCR. In addition, six patients with breast cancer requiring neoadjuvant chemotherapy (NACT) also underwent functional MRI examinations and IHC to determine potential antitumor immune response.

**Results:**

At the end of the study, the CPA treatment group showed the lowest tumor volume compared to the control group. For pathological examinations, the CPA treatment group showed a lower percentage of CD31 staining (*P* < 0.01) and Ki-67 staining (P<0.01), and a higher percentage of TUNEL staining (*P* < 0.01). The tumoral pseudodiffusion coefficient (D*) value showed a positive correlation with the CD31-positive staining rate (*r* = 0.729, *P <* 0.0001). The diffusion related parameters (D) value was positively correlated with TUNEL (r = 0.858, *P <* 0.0001) and negatively correlated with Ki-67 (r = -0.904, *P <* 0.0001). Moreover, a strong induction of the expression of the immune responses in the CPA treatment group was observed on day 12. D values showed a positive correlation with the Ifnb1-, CD8a-, Mx1-, Cxcl10- (*r* = 0.868, 0.864, 0.874, and 0.885, respectively, *P* < 0.0001 for all). Additionally, the functional MRI parameters and IHC results in patients with breast cancer after NACT also showed a close correlation between D value and CD8a (*r* = 0.631, *P* = 0.028).

**Conclusions:**

The treatment response induced by immunogenic chemotherapy could be effectively evaluated using IVIM-DWI. The D values could be potential, sensitive imaging marker for identifying the antitumor immune response initiated by immunogenic chemotherapy.

## Introduction

Conventional cytotoxic cancer chemotherapy is often immunosuppressive and related to drug resistance and tumor regrowth (1–3). However, traditional chemotherapy drugs, including doxorubicin, mitoxantrone, and cyclophosphamide(CPA), potentially increase the immunogenicity of tumor cells by activating immunogenic cell death (ICD), thereby activating innate immune responses and eliciting tumor-specific adaptive immune responses, and has the potential to greatly improve the efficacy of chemotherapy (4–6). Neoadjuvant chemotherapy (NACT) or primary systemic therapy is considered the standard treatment for locally advanced breast cancer. The accumulation of preclinical and clinical evidence indicates that the success of anthracycline-based NACT for breast cancer depends (at least in part) on their ability to stimulate anticancer immune responses (7–9). High levels of tumor-infiltrating lymphocytes (TILs) are predictive of complete pathological response after neoadjuvant chemotherapy in certain breast cancers.

Immunogenic chemotherapy is a regimen characterized by regular and frequent chemotherapeutic drug doses that maintain a low but active antitumor immune response during prolonged periods without significant toxicity. Immunogenic chemotherapy can inhibit tumor angiogenesis, stimulate the antitumor immune response, and induce tumor dormancy (10). A study revealed that medium-dose, intermittent chemotherapy (MEDIC) of CPA could induce complete immune cell-dependent regression of GL261 tumors implanted in immune-competent C57BL/6 mice in a GL261(B6) tumor model and activate long-term tumor-specific immunity (11). Specifically, MEDIC chemotherapy of CPA could stimulate the production and release of type-I interferons and lead to the robust activation of downstream gene targets, including Mx1 and Cxcl10 (11). These tumor-associated gene responses are linked to innate immune cell recruitment and tumor regression.

In clinical practice, monitoring the antitumor immune response is critical and may convey robust predictive or prognostic indications (12, 13). However, clinical challenges remain in identifying those patients most likely to respond and accurately monitoring the clinical response when patients receive antitumor treatment. Although the pathological examination is the gold standard for detecting the antitumor immune response, it is invasive and cannot be performed repetitively due to the potential risk of infection and metastasis. Therefore, it is worth having a reliable and noninvasive biomarker to identify and monitor the antitumor immune response.

Intravoxel incoherent motion diffusion-weighted imaging (IVIM-DWI), a promising functional MRI imaging technique, could evaluate imaging features of the tumor and assess the therapeutic efficacy after radio/chemotherapy in many tumors (14). However, there are still no studies that assess the immune response induced by anti-neoplastic agents using IVIM-DWI. Hence, this study aims to investigate the utility of IVIM DWI in detecting the immune response induced by MEDIC chemotherapy of CPA in a GL261 mouse glioma model and induced by neoadjuvant chemotherapy in patients with breast cancer.

## Material and Methods

### Animal Model and Treatments

Our animal operations were performed following a protocol approved by our institutional animal care and use committee. The GL261 murine glioblastoma cells was obtained from the DSMZ, Germany. Six-week-old male C57BL/6 (B6) mice were provided by Beijing Vital River Laboratory Animal Technology Co., Ltd. (Beijing, China). A total of 5 x 10^6^ GL261 cells were suspended in 0.2-mL serum-free RPMI per site and subcutaneously injected into the posterior flanks of B6 mice to develop the subcutaneous glioma tumor model.

The tumor volume was measured every two days using Vernier calipers and was calculated as Vol D (π/6)* (L * W)^3/2^ (L=length,W=width). The mice in the treated group (n = 14) were intraperitoneally administered CPA monohydrate (Cat. # C0768, Sigma-Aldrich, St. Louis, MO) at a dose of 140 mg/kg-body weight per injection (CPA-140); The control group (n=5) received an intraperitoneal injection of saline at the same dose. All mice were treated by intraperitoneal injection on days 0 and 6.

### MR Imaging

MR imaging (MRI) was performed on a 3.0-T MRI system (Signa HDxt, GE Medical System, Milwaukee, WI, USA) with a custom-built 8-channel receiver coil with a 3 cm inner diameter (Shenzhen Teshen Electric Co., Ltd., Shengzhen, China). The two groups of mice were intraperitoneally administered with 0.1% pentobarbital solution and scanned in the prone position before treatment (day 0, baseline) and on days 6 and 12 after CPA treatment or saline. The B6 mice with GL261 were covered with a thin slice of pork to reduce the distortion MRI artifact. After routine localization images were obtained, Transverse T2-weighted images (T2WI) and T1-weighted images (T1WI) were obtained using a fast spin-echo (FSE) sequence: TR = 3000.0/625.0ms, TE = 68.0/9.4ms, slice thickness= 1.6/1.6 mm, slice gap = 0.1/0.1 mm, field of view (FOV) = 10×10/10×10 cm, matrix size = 288×288/288×288, and NEX=4/2. IVIM-DWI MRI was acquired using single-shot echo-planar imaging (TR=3000ms, TE=102.4ms, slice thickness=2.9 mm, slice gap=0.1 mm, matrix size=128×64, and FOV=10×6cm) with diffusion gradients applied in three orthogonal directions (12 b values=0, 20,50, 100, 150, 200, 400, 600, 800, 1200, 1600, 2000s/mm^2^, NEX = 4). The scanning time of IVIM-DWI was 6 minutes 45s.

### Imaging Analysis

For analysis, all the acquired MR images were transferred to a postprocessing workstation (ADW4.5, GE Healthcare). The parametric maps of IVIM-fMRI were generated automatically by Functool-MADC software. The IVIM-derived parameter was calculated by equations (1) and (2), which were described by Le Bihan et al. (15, 16).


(1) Sb/S0=(1−f) exp (−bD)

(2) Sb/S0=(1−f) exp (−bD)+f exp (−bD* )

Sb/S0 stands for the signal intensity of the corresponding diffusion gradient b or 0 values. The D values represent the true diffusion coefficient, D* represents the pseudodiffusion coefficient, and f is the perfusion fraction. The blood flow-related parameter (fD*) was calculated by multiplying f by D* (17). The values of all IVIM-DWI parameters were measured independently by two radiologists with five years of experience in MRI. The descriptive statistics of the D, D*, and f value of tumors among groups of high b-value distribution are shown in **Tables S1, S2**, and **Figures S1-S3** (**Supplementary Information**); we finally excluded b values greater than 1000s/mm^2^ and set the b-value distribution to 0-800s/mm^2^ when performing the IVIM study to make the measurement more accurate (18, 19).

We delineated the tumor border as regions of interest (ROIs) on the largest cross-section of the tumor. The ROI was then copied to the pseudocolor maps at the same cross-section of the tumor, including D, D*, and f, to obtain the parametric values. The average of three ROI values on the three largest cross-sections of the tumor was used as a representative parametric value.

### Histological Assessment and Quantitative Real-Time PCR

After the last scan (on day 12), tumors were excised for quantitative PCR(qPCR) and immunohistochemistry. qPCR was performed with an SYBR Premix Ex Taq Kit (Takara, Japan) in a CFX96 Touch Real-Time PCR System (Bio-Rad, USA) for marker gene analysis, including cytotoxic T-cells (CD8a), type-I interferons(Ifnb1), ISGs Mx1 and Cxcl10.

A portion of the tumor was fixed in 4% paraformaldehyde, embedded in paraffin, and cut into three μm thick sections for hematoxylin and eosin (H&E) staining, Ki67, CD8a, CD31, and terminal deoxynucleotidyl transferase-mediated dUTP nick end labeling (TUNEL) stains. Ki67, CD8a, CD31, and Tunel were conducted to assess cell proliferation, tumor-infiltrating lymphocytes, angiogenesis, and apoptosis levels. All antibodies were purchased from Boster Bioengineering Co., Ltd. (Wuhan, China). Staining intensity (0 to 3+) and extent (0%to 100%) were scored for all tumors with an H-score calculated (range, 0 to 300) using Image-Pro Plus 6.0 software (Media Cybernetics, MD, USA).

### Study Population

We enrolled six consecutive patients who had been scheduled for NAC from June 2020 to July 2020. All patients met the following criteria: (1) unilateral invasive ductal carcinoma confirmed by needle biopsy before NAC; (2) routine MRI and IVIM-DWI scans were performed before NAC, after cycle one (in the first three days of cycle two), and after cycle two (in the first three days of cycle two); (3) no surgery, chemo/radiotherapy, hormone therapy or any other treatment before the first MR examination; (4) no evidence of distant metastases before NAC; and (5) surgery at our hospital within three weeks after the completion of NAC.

### Chemotherapy Regimens

The Chemotherapy regimen was composed of a TAC regimen (docetaxel, cyclophosphamide, pirarubicin) for four patients: and another TAC regimen (docetaxel, epirubicin, cyclophosphamide) for two patients.

### Imaging Technique

MR imaging (MRI) was performed on a clinical 3.0-T MRI system (Signa HDxt, GE Medical System, Milwaukee, WI, USA) with a 4-channel breast coil. The subject was asked to lie in the prone position, with the breasts naturally falling in the coil.

Horizontal T2 fat suppression images (repetition time/echo time, 3570/72ms; section thickness, 5.0 mm; matrix, 256× 230) and horizontal T1 fat suppression images (repetition time/echo time, 169.0/92.61ms; section thickness, 4.0 mm; matrix, 448×380) were acquired.

The IVIM-DWI sequence was generated using single-shot echo-planar imaging with 12 b-values of 0, 20, 50, 100, 150, 200, 400, 600, 800, 1200, 1600, and 2000 s/mm^2^. The following parameters were used for this sequence: TR/TE, 6400 ms/63.0 ms; flip angle, 90°; matrix, 192 × 192; field of view, 340 mm ×136 mm; section thickness, 5.0 mm; NEX, 4. The steps for imaging analysis and quantitative measurement of IVIM parameter maps were the same as those described above.

The steps for IHC analyses of the patients were the same as those described above.

### Statistical Analysis

The SPSS 16.0 software (IBM Corporation, Chicago, IL, USA) and GraphPad Prism 7.01 (GraphPad Software Inc., San Diego, CA) were utilized to perform statistical tests and plot line charts. The quantitative results are expressed as medians and ranges.

For comparing the control and treated groups in glioma-bearing mice, the Mann-Whitney test was used to analyze the median values of IVIM-DWI parameters and all histopathological indices of the gliomas in C57BL/6 mice. The intraclass correlation coefficient (ICC) with 95% confidence intervals (CI) (two-way random model, absolute agreement, and single measure) was calculated to assess the interobserver agreement of the MRI parameters. ICC values were interpreted as follows: poor, < 0.5; moderate, 0.5-0.75; good, 0.75-0.9; excellent, 0.9-1.0 (20). Spearman’s rank correlation test was performed for correlations between histological features and the corresponding IVIM-DWI parameters. The Kruskal-Wallis test was used to analyze changes in the IVIM-DWI parametric values in breast cancer patients receiving NACT. An *r ≥* 0.8 was considered highly correlated, whereas *r* < 0.8 and *r ≥* 0.5 were considered mildly correlated. A *P* value < 0.05 was considered statistically significant.

## Results

### Effect of CPA Treatment on Tumor Growth

**Figure 1:**
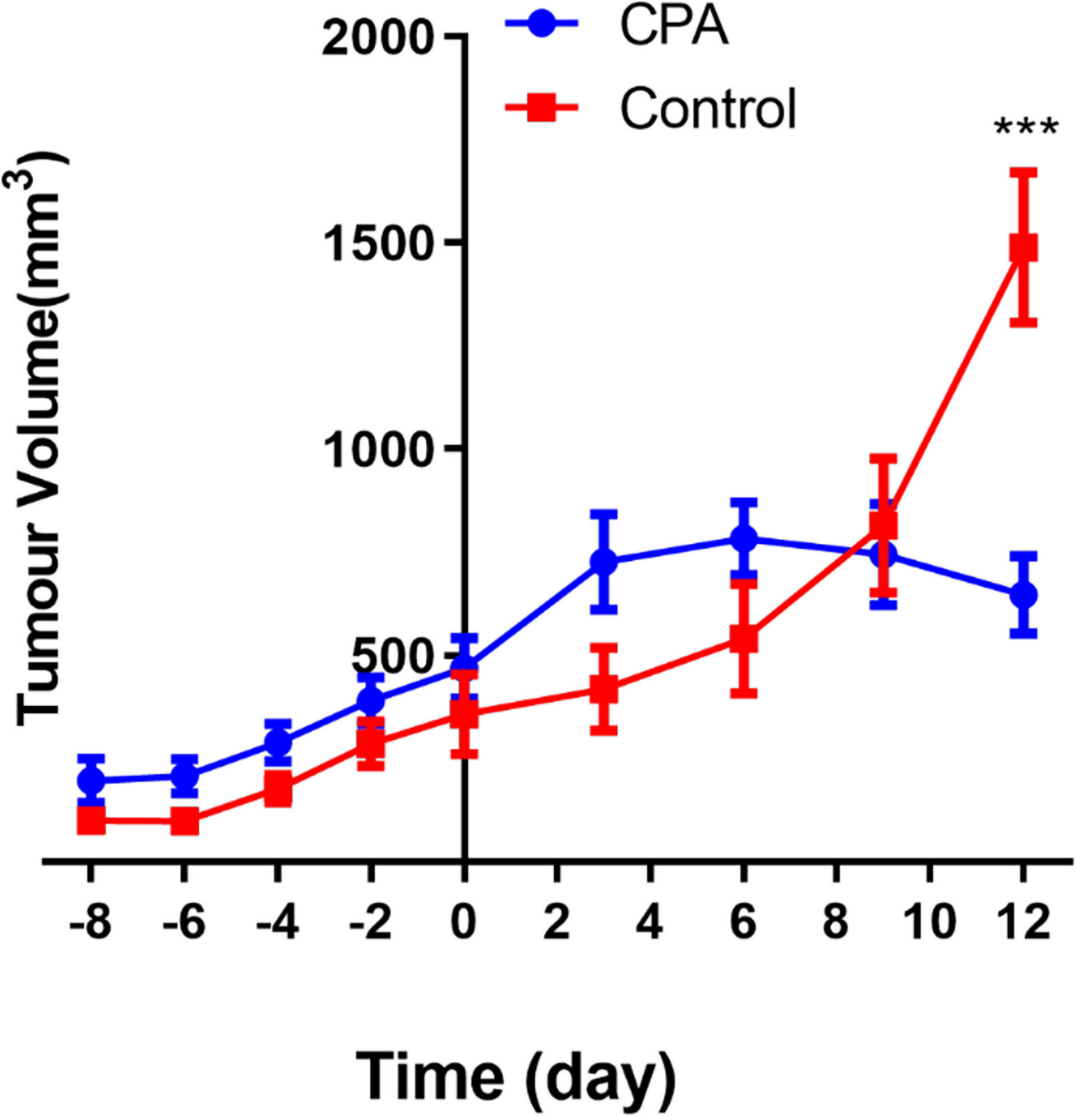
Longitudinal assessment of the tumor volume in the two groups. The data are presented as the mean ± standard deviation. ***P < 0.001 refers to comparisons between groups at corresponding time points using the two-independent-samples t-test.

MEDIC CPA treatment for 12 days effectively inhibited the tumor growth of GL261 gliomas. As shown in **Figure 1**, the tumor volume of the treated group began to decrease on day 6 after CPA treatment, while that of the control group continued to increase. The tumor volume in the treated group was significantly lower than that in the control group on the 12th day (P < 0.001).

### Treatment Efficacy Evaluated by MRI

Moderate agreement between the two experienced radiologists was found for the D* value (ICC:0.693, 95%CI:0.360 to 0.869); the agreements for the the D and f values were good (ICC:0.963, 95%CI:0.906 to 0.985, and ICC:0.795, 95%CI:0.623 to 0.914, respectively).

**Figure 2:**
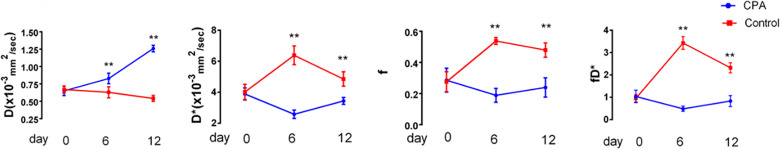
Longitudinal assessments of the MRI parameters in the CPA and control groups. Data points are plotted as medians and ranges. **P < 0.01 refers to comparisons between groups at corresponding time points using the Wilcoxon rank-sum test.

**Figure 3:**
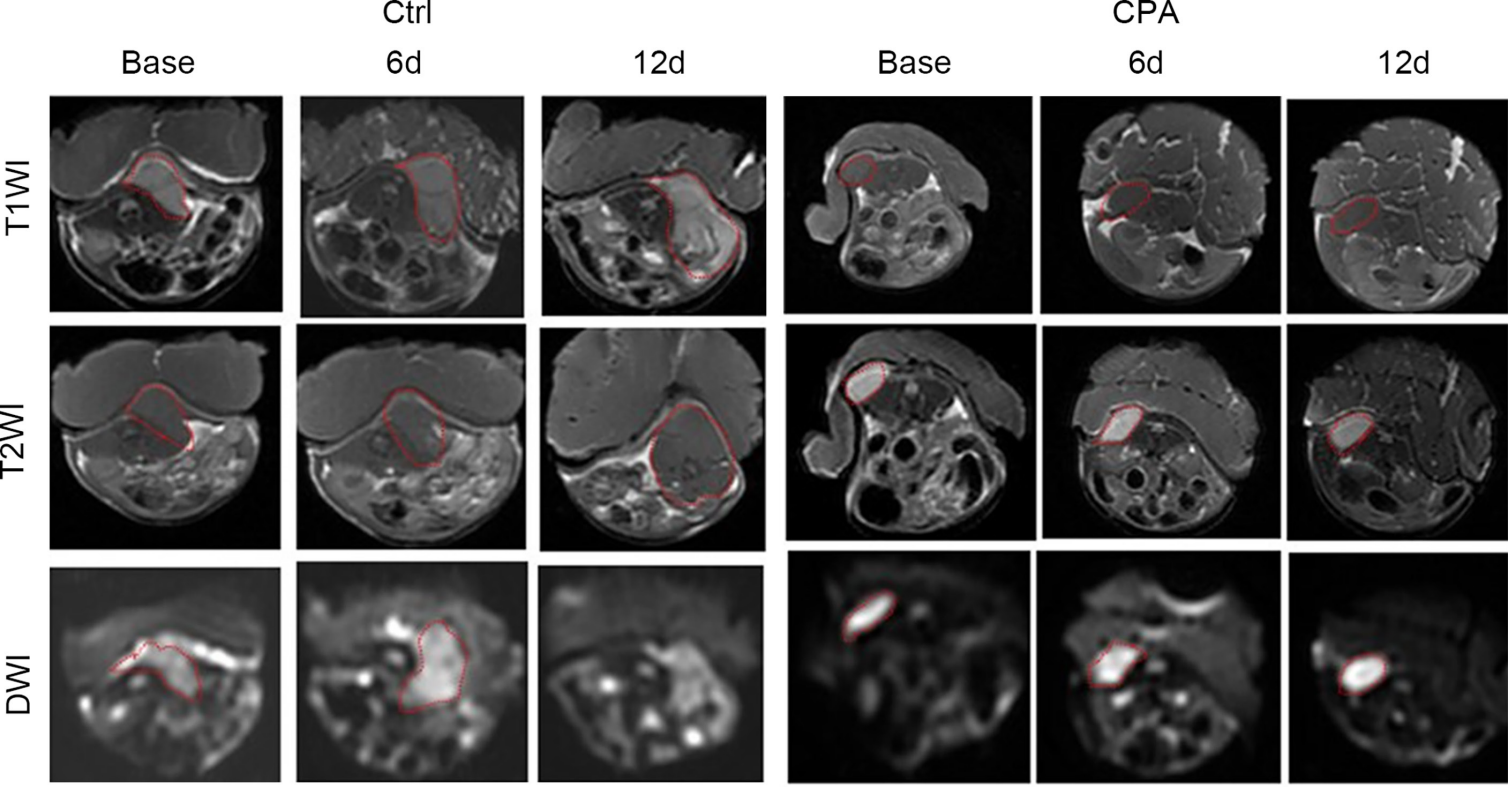
Conventional MRI (T1- and T2-weighted images) and functional maps of representative tumors in mice before and at different time points after treatment in the control and CPA-treated groups. Dashed outlines denote tumor areas.

To dynamically monitor perfusion and water diffusion changes in the tumor microenvironment, we performed IVIM-DWI on the two groups longitudinally. At baseline, there was no significant difference in any IVIM-DWI parameters between the control and treated groups. The tumoral D values in the treated group were significantly greater than those in the control group on both days 6 and 12 (P<0.005). In contrast, the D*, f, and fD* values in the treated group were significantly lower than those in the control group at the same time points (P<0.005) (**Figures 2**, **3**).

In the treated group, the D*, f, and fD* values significantly decreased on day 6 compared with those at baseline and then increased to near baseline levels on day 12. However, those values showed the opposite trend in the control group. The D values in the treated group demonstrated significantly consistent growth from baseline to day 12 after CPA treatment. In contrast, those in the control group gradually decreased (**Table 1**).

**Table 1:** Comparisons of the IVIM-DWI parameters between the CPA group and control group of the GL261 model.

Parameter	Baseline	D_6_	D_12_
**D(× 10^-3^ *mm* ^2^/s)**			
Control	0.66 (0.09)	0.62 (0.14)	0.52 (0.07)
CPA	0.66 (0.09)	0.86 (0.23)	1.26 (0.08)
P value	P=0.711	P=0.003**	P=0.001**
D*(× 10^-3^ *mm* ^2^/s)			
Control	3.97 (0.75)	6.30 (1.20)	4.80 (0.78)
CPA	3.82 (0.59)	2.56 (0.46)	3.53 (0.38)
P value	P=0.578	P=0.001**	P=0.001**
***f***			
Control	0.21 (0.04)	0.55 (0.04)	0.49 (0.08)
CPA	0.29 (0.06)	0.18 (0.06)	0.23 (0.06)
P value	P=0.137	P=0.001**	P=0.001**
fD*(× 10^-3^ *mm* ^2^/s)			
Control	1.00 (0.28)	3.25 (0.51)	2.36 (0.38)
CPA	1.02 (0.29)	0.47 (0.17)	0.77 (0.31)
P value	P=0.579	P=0.001**	P=0.003**

The data are the median, and the interquartile range is in parentheses. The Mann-Whitney U test was used to compare the differences in IVIM-DWI parameters between the two groups. **P < 0.01 represents the analysis results. ^##^D* represents the pseudodiffusion coefficient; fD*represents the blood flow-related parameter.

### Treatment Efficacy Assessed by Histopathology

**Figure 4:**
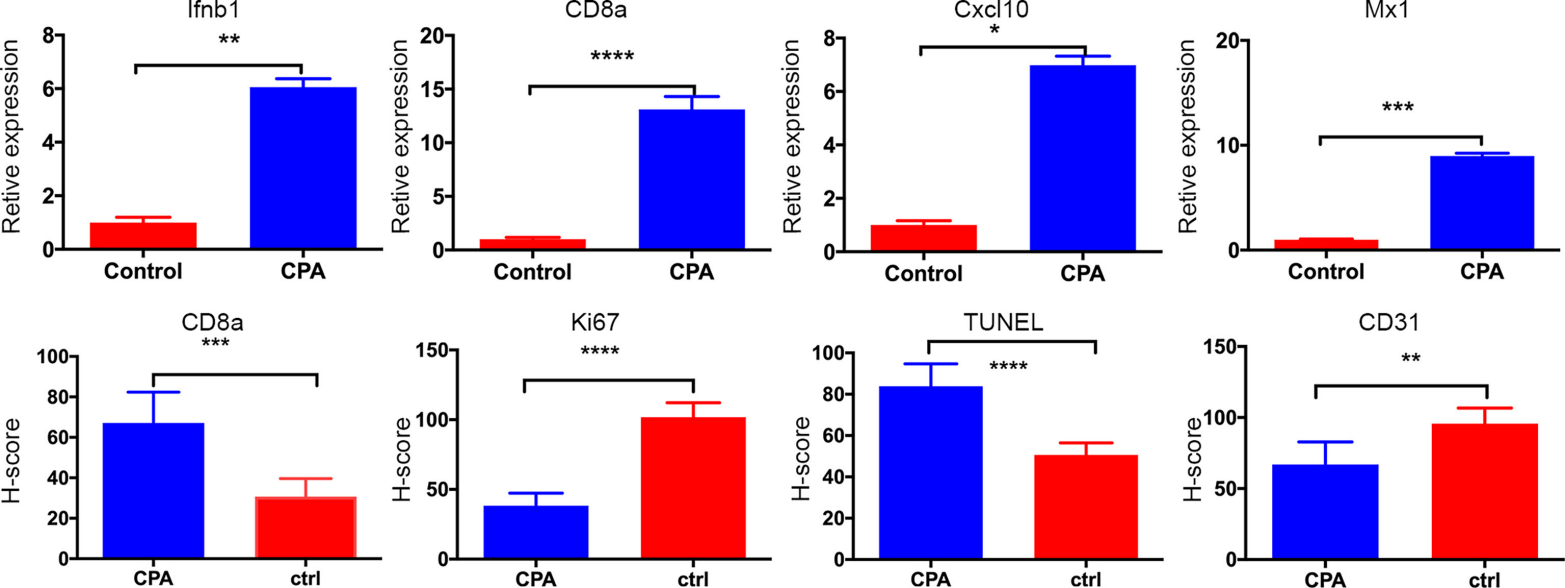
Quantitative results of pathological indicators in the CPA and control groups. The data are presented as the mean ± standard deviation. *P < 0.05, **P <0.01, ***P < 0.001 and ****P < 0.0001 refer to comparisons between groups on day 12 using the two-independent-samples t-test.

**Figure 5:**
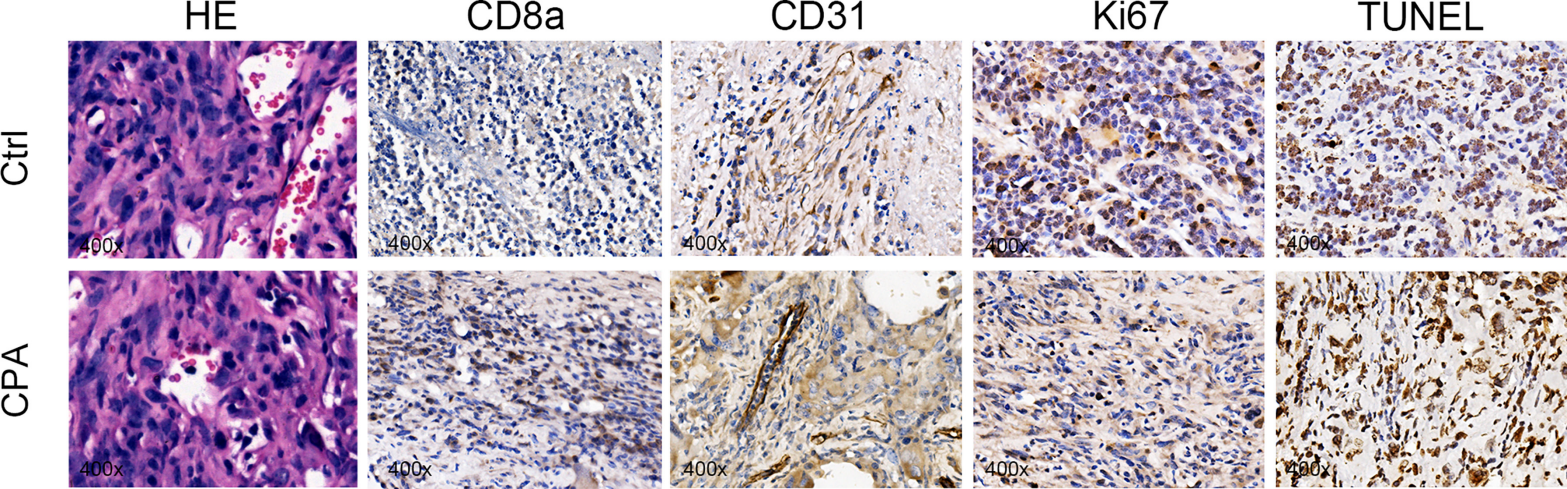
Representative pictures of HE (×400), CD8a (×400), CD31 (×400), Ki67 (×400), and TUNEL staining (×400) between groups on day 12 after treatment.

After the experiment, we performed CD31, TUNEL, and Ki-67 assays to evaluate angiogenesis, tumor cell apoptosis, and proliferation, respectively. The CPA-treated group showed a significantly lower percentage of CD31 staining and Ki-67 staining and a significantly higher percentage of TUNEL staining compared to the control group (*P <*0.01 for all) (**Figure 4**). Representative pathologically stained sections of the two groups were presented in **Figure 5**.

Tumor regression was associated with a significant increase in immune cell infiltration, as revealed by the marker gene analysis in the completely excised GL261 tumors on day 12. Immune cell recruitment and expression in the CPA-treatment group were significantly higher than those in the control group. A massive increase in cytotoxic T-cells (CD8a) and the expression of type-I interferons were observed in the CPA group (*P* < 0.01 for all). Moreover, strong induction of the ISGs *Mx1* and *Cxcl10* was also observed (*P* < 0.05 for all) (**Figure 4**).

### Correlations Between Functional MRI Parameters and Pathologic Markers and Immune Response Markers

For pathological examinations, the perfusion-related parameters D* value showed a positive correlation with the CD31-positive staining rate (*r* = 0.729, *P<* 0.0001). The diffusion related parameters (D) value was positively correlated with TUNEL (*r* = 0.858, *P <* 0.0001) and negatively correlated with Ki-67 (*r* = -0.904, *P <* 0.0001).

Moreover, a strong induction of the expression of the immune responses in the CPA treatment group was observed on day 12. D values showed a positive correlation with the Ifnb1-, CD8a-, Mx1-, Cxcl10-(*r* = 0.868, 0.864, 0.874, and 0.885, respectively, *P* < 0.0001 for all)

### Treatment Efficacy Evaluated by MRI and IHC in Breast Cancer Patients After NACT

**Figure 6:**
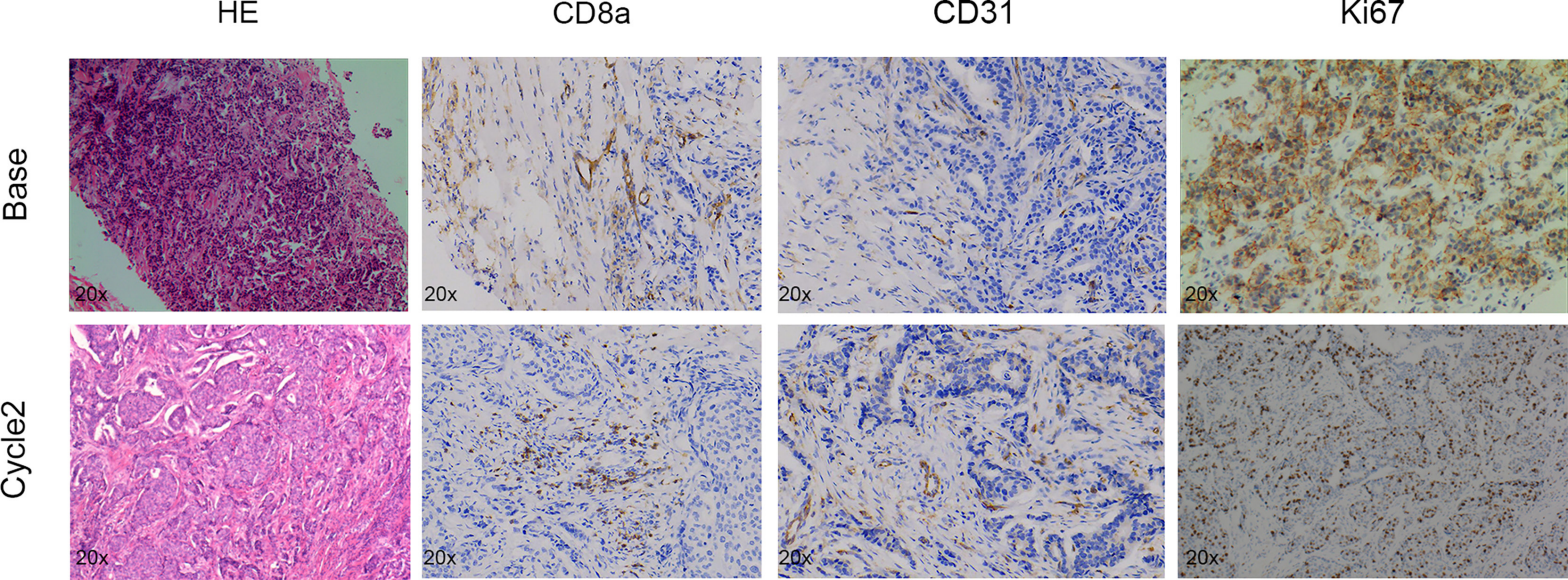
Pathologic tumor changes after neoadjuvant chemotherapy treatment in breast cancer. Representative HE (×20), CD8a (×20), CD31 (×20), and Ki67 (×20) at baseline and time points after two cycles of treatment.

**Figure 7:**
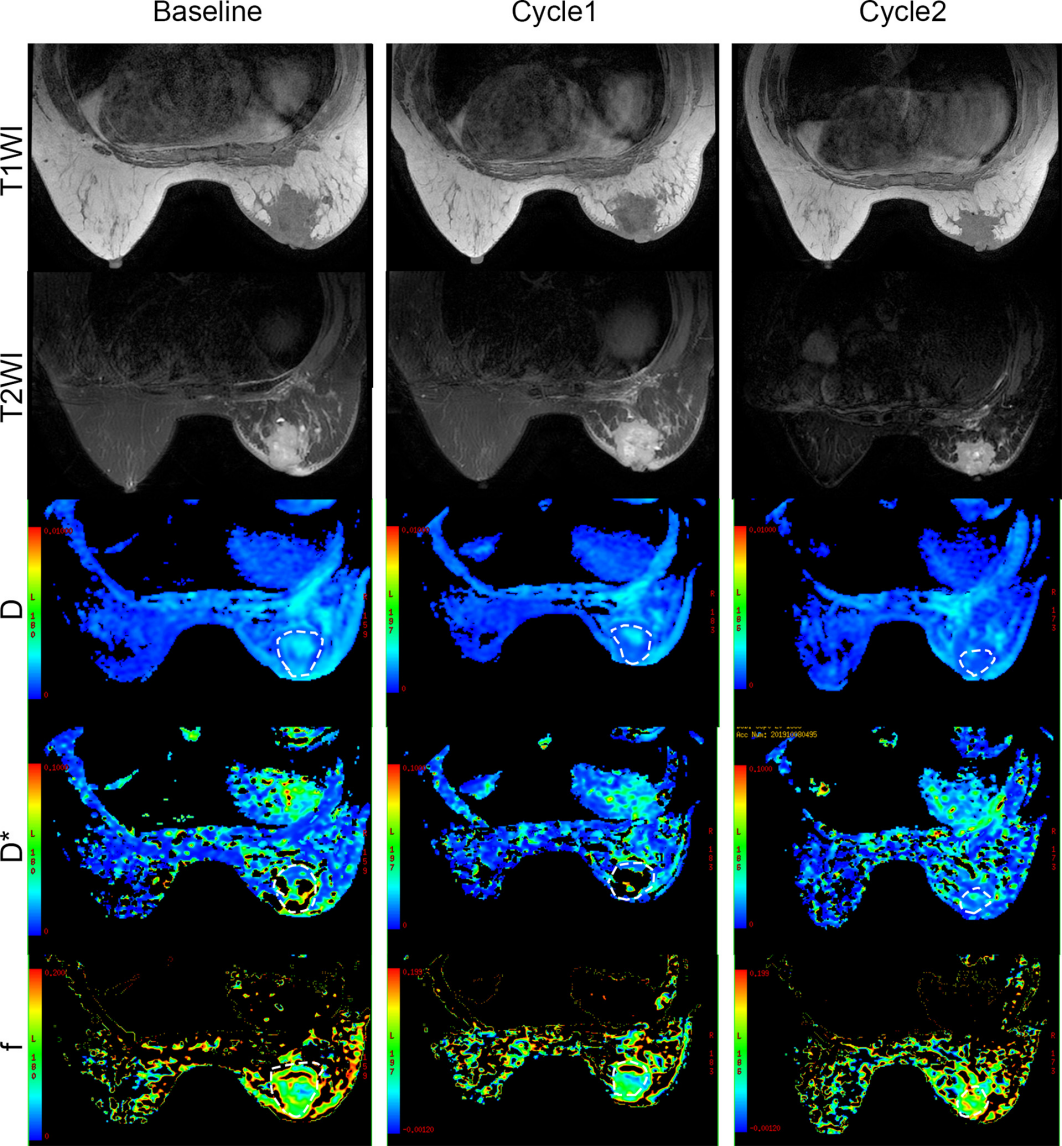
Conventional MRI (T1- and T2-weighted images) and functional maps in breast cancer patients with two cycles of immunochemotherapy. Dashed outlines denote tumor areas. Color ranging from blue to red represents values ranging from low to high.

To monitor the antitumor response after neoadjuvant therapy in patients with breast cancer, we conducted IVIM-DWI and IHC in these six patients longitudinally. The perfusion-related D*, f, and fD* values gradually decreased with statistically significant differences. However, the diffusion-related D values demonstrated significantly consistent growth (**Table 2** and **Figures 6**, **7**). The D value showed a positive correlation with CD8a (*r* = 0.631, *P* = 0.028) and a negative correlation with Ki67 (*r* = -0.733, *P* = 0.007). Positive correlations were found between the D* values and CD31 (*r* = 0.776, *P* = 0.003).

**Table 2:** Comparisons of IVIM-DWI parameters in breast cancer patients with 2 cycles of neoadjuvant chemotherapy.

Parameter	Baseline	Cycle1	Cycle2	H	P
**D(× 10^-3^ *mm* ^2^/s)**	0.58 (0.47-0.66)	0.84 (0.70-0.99)	1.11 (0.75-1.40)	8.77	0.012
D*(× 10^-3^ *mm* ^2^/s)	10.35 (10.2-14.0)	7.22 (5.1-8.49)	6.63 (3.99-7.99)	7.73	0.021
***f***	0.34 (0.27-0.38)	0.19 (0.16-0.27)	0.11 (0.064-0.13)	9.27	0.01
fD*(× 10^-3^ *mm* ^2^/s)	3.60 (2.88-5.17)	1.18 (1.0-2.23)	0.80 (0.29-0.98)	9.27	0.01

The data are the median, and the interquartile range is in parentheses. The Kruskal-Wallis test was used to compare the differences in IVIM-DWI parameters. D* represents the pseudodiffusion coefficient; fD* represents the blood flow-related parameter.

## Discussion

Certain cytotoxic drugs have immunogenic properties that can promote the activation of the immune system, and many pre-clinical and clinical evidence support the importance of the immune cell infiltrate in the prognosis of tumors (4, 21, 22). Given the importance of monitoring antitumor immune responses in clinical practice, the purpose of our study was to evaluate the predictive value of IVIM DWI for the quantitative assessment of immune responses activated by conventional chemotherapy in mouse models and clinics. We successfully built a mouse glioma model with induced antitumor immune responses. By measuring MRI parameters at each time point during treatment, we dynamically assessed the changes in the tumor microenvironment. Interestingly, we found that the D values were strongly correlated with markers of the chemotherapy-related antitumor immune response. Moreover, we collected MRI data and IHC results in patients with locally advanced breast cancer requiring neoadjuvant chemotherapy. The results showed that preclinical findings were consistent with clinical observations.

The IVIM-DWI parametric values indicated unrestricted water molecule diffusion and decreased perfusion in glioma after CPA treatment related to the antitumor responses induced by CPA. These changes can be explained by histologic findings. On day 12, Ki67 was significantly reduced, and TUNEL was significantly increased in the CPA-treated group, suggesting a decrease in tumor cell density (23, 24). The diffusion-related parameter D is the pure diffusion of water molecules in the extracellular space, related to the cell density. Our results showed that the diffusion-related parameter D was negatively correlated with Ki-67 and positively correlated with TUNEL, respectively. When the cell density decreases and the extracellular space expands, the movement of water molecules is unrestricted, resulting in higher D values. The perfusion-related D* values were positively correlated with CD31 that were significantly reduced in the CPA-treated group on day 12, indicating that angiogenesis of tumors was inhibited.

In addition, we found strong (or robust) induction of immune response expression in the CPA treatment group, and the D value was positively correlated with CD8a, Ifnb1, Mx1, and Cxcl10 expression. The findings may be related to the large production and release of Ifnb1 induced by CPA chemotherapy. Ifnb1 has a potent inhibitory effect on tumor cell proliferation and immune regulation. First, it promotes macrophages to swallow antibody-coated tumor cells, activates NK cells, and enhances cytotoxicity. In addition, it can inhibit the proliferation of tumor cells (25) and promote tumor cell apoptosis (26). These factors will cause necrosis and fibrosis in tumor cells, reduce their internal density, expand the extracellular space, and increase water molecules’ diffusion. Thus, the D value could be a sensitive and noninvasive predictor in monitoring the antitumor immune response.

Moreover, our results showed that preclinical findings were consistent with clinical observations. We collected MRI data and IHC results from 2 cycles of 6 patients with locally advanced breast cancer who required neoadjuvant chemotherapy. The D*value gradually decreased and were associated with CD31; the D value demonstrated a significantly consistent increase and were related to cell apoptosis and immune response. Neoadjuvant therapy for cancer is any anticancer treatment provided before the main treatment (usually surgery) and thus constitutes a form of induction therapy (27). In breast cancer, neoadjuvant chemotherapy is the preferred treatment approach for locally advanced cancers with a pathologic complete response (28). Chemotherapy has long been considered immune suppressive; however, there is growing evidence that the efficacy of chemotherapy involves cell-intrinsic cytotoxic effects and relies on the activation of antitumor immune responses (2). Recently, a study confirmed that after neoadjuvant chemotherapy for breast cancer, just one cycle of treatment could induce an immune stimulatory microenvironment and upregulation of inflammatory signatures (29). This finding may explain why the IVIM-DWI parameters follow the same trend in animal experiments and clinical trials.

Several factors constrained this exploratory study. First, only three-time points were set to evaluate the antitumor immune response induced by CPA treatment. The increased time points could provide additional information about the dynamic variation of tumoral IVIM-DWI parameters for monitoring the antitumor immune response induced by CPA. Second, the b-values of IVIM-DWI may be too high (up to 2000s/mm^2^), which may bring difficulties in interpreting IVIM-DWI data. Finally, the small sample size is also a major limitation in our study, which may increase Type II errors.

In conclusion, our results suggest that conventional chemotherapy can activate the anti-tumor immune response. IVIM-DWI can be used to monitor the changes in the tumor microenvironment in a noninvasive manner. In addition, the D values could be potential, sensitive imaging markers for identifying the antitumor immune response.

## Data Availability Statement

The original contributions presented in the study are included in the article/**Supplementary Material**. Further inquiries can be directed to the corresponding authors.

## Ethics Statement

The studies involving human participants were reviewed and approved by the Ethics Committee of the First Affiliated Hospital of Jinan University. The patients/participants provided their written informed consent to participate in this study. The animal study was reviewed and approved by Institute of Laboratory Animal Science, Jinan University. Written informed consent was obtained from the owners for the participation of their animals in this study. Written informed consent was obtained from the individual(s) for the publication of any potentially identifiable images or data included in this article.

## Author Contributions

JH carried out the studies, participated in collecting data, and drafted the manuscript. JH, XY, and PY performed the statistical analysis and participated in its design. XC and BD reviewed and helped to draft the manuscript. All authors contributed to the article and approved the submitted version.

## Funding

This study has received funding by Natural Science Foundation of Guangdong Province (grant no.2017A030313901), Guangzhou Science, Technology and Innovation Commission (grant no.201804010239).

## Conflict of Interest

The authors declare that the research was conducted in the absence of any commercial or financial relationships that could be construed as a potential conflict of interest.

## Publisher’s Note

All claims expressed in this article are solely those of the authors and do not necessarily represent those of their affiliated organizations, or those of the publisher, the editors and the reviewers. Any product that may be evaluated in this article, or claim that may be made by its manufacturer, is not guaranteed or endorsed by the publisher.

## Abbreviations

CPA, cyclophosphamide; MEDIC, medium-dose intermittent chemotherapy; RT-qPCR, reverse transcription-quantitative polymerase chain reaction; IFN, interferon; ISG, IFN-stimulated gene; ICC, intraclass correlation coefficient; D, true diffusion coefficient; D*, pseudodiffusion coefficient; DW, diffusion weighted; f, perfusion fraction; fD*, blood flow-related parameter; IVIM, intravoxel incoherent motion; NACT, Neoadjuvant chemotherapy.
