# Supplementary material for: Intravoxel Incoherent Motion Diffusion-Weighted MR Imaging for Monitoring the Immune Response of Immunogenic Chemotherapy
Source: Front Oncol. 2022 May 13;12:796936. doi: 10.3389/fonc.2022.796936 (PMC9136146; doi:10.3389/fonc.2022.796936)
Supplement: Supplementary file 1 [file DataSheet_1.docx]

| Parameter | baseline | Day _6_ | Day _12_ |
| --- | --- | --- | --- |
| **D(×**$\boldsymbol{10}^{\boldsymbol{-3}}\boldsymbol{mm}^{\boldsymbol{2}}$**/s)** | | | |
| Group 1 | 0.65± 0.07 | 0.83 ± 0.08 | 1.26 (1.21-1.29) |
| Group 2 | 0.55 ± 0.09 | 0.65 ± 0.03 | 0.89 (0.83- 0.92) |
| *t/Z* | 3.34 | 6.53 | -4.51 |
| *P* value | 0.003 | *P*< 0.001 | *P*< 0.001 |
| ***D**(×**$\boldsymbol{10}^{\boldsymbol{-3}}\boldsymbol{mm}^{\boldsymbol{2}}$**/s)** | | | |
| Group 1 | 3.89 ± 0.39 | 2.58 ± 0.27 | 3.53(3.20- 3.58) |
| Group 2 | 1.71 ± 0.38 | 1.38 ± 0.13 | 1.27 (1.14- 1.62) |
| *t/Z* | 14.75 | 15.20 | -4.50 |
| *P* value | *P*< 0.001 | *P*< 0.001 | *P*< 0.001 |
| ***f* (%)** | | | |
| Group 1 | 0.29 ± 0.0.08 | 0.19 ± 0.04 | 0.24 ± 0.06 |
| Group 2 | 0.55 ± 0.04 | 0.38 ± 0.05 | 0.54 ± 0.03 |
| *t* | -11.68 | -11.35 | -16.28 |
| *P* value | *P*< 0.001 | *P*< 0.001 | *P*< 0.001 |

**SUPPLEMENTARY INFORMATION**

**Table S1 Comparisons of the IVIM-DWI parameters between the Group 1（b= 0-800 s/mm^2^ and Group 2（b= 0-2000s/mm^2^）of the treated group**

Note: Comparisons were performed with the Student's t-test (for normally data), or Mann-Whitney U test (for nonparametric data).

**Table S2 Comparisons of the IVIM-DWI parameters between the Group 1（b= 0-800**

**s/mm^2^ and Group 2（b= 0-2000s/mm^2^）of the control group**

| Parameter | baseline | Day _6_ | Day _12_ |
| --- | --- | --- | --- |
| **D(×**$\boldsymbol{10}^{\boldsymbol{-3}}\boldsymbol{mm}^{\boldsymbol{2}}$**/s)** | | | |
| Group 1 | 0.67 ± 0.05 | 0.62 ± 0.08 | 0.52 (0.51-0.58) |
| Group 2 | 0.54 ± 0.02 | 0.51 ± 0.06 | 0.42 (0.42- 0.43) |
| *t/Z* | 4.83 | 2.51 | -2.66 |
| *P* value | 0.001 | 0.036 | 0.008 |
| ***D**(×**$\boldsymbol{10}^{\boldsymbol{-3}}\boldsymbol{mm}^{\boldsymbol{2}}$**/s)** | | | |
| Group 1 | 4.02 ± 0.49 | 5.58 ± 0.39 | 4.25 ± 0.38 |
| Group 2 | 1.89 ± 0.32 | 2.67 ± 0.21 | 1.6 ± 0.09 |
| *t* | 8.1 | 14.4 | 15.2 |
| *P* value | *P*< 0.001 | *P*< 0.001 | *P*< 0.001 |
| ***f* (%)** | | | |
| Group 1 | 0.28 ± 0.06 | 0.54 ± 0.02 | 0.48 ± 0.05 |
| Group 2 | 0.53 ± 0.02 | 0.68 ± 0.04 | 0.64 ± 0.02 |
| *t* | -8.5 | -6.5 | -6.94 |
| *P* value | *P*< 0.001 | *P*< 0.001 | *P*< 0.001 |

Note: Comparisons were performed with the Student's t-test (for normally data), or Mann-Whitney U test (for nonparametric data).

**Figure S1** Correlations of each IVIM parameter between the two groups at each time point

**Figure** **S2** The relationship between D values and the expressions of immune-related genes in each group

**TUNEL(%)**

**Ki67**

**Figure S3** The relationship between D and D* values and the results of immune-histochemistry in each group
